# Transcription factor KLF15 inhibits the proliferation and migration of gastric cancer cells via regulating the TFAP2A-AS1/NISCH axis

**DOI:** 10.1186/s13062-021-00300-y

**Published:** 2021-11-03

**Authors:** Xin Zhao, Linlin Chen, Jingxun Wu, Jun You, Qingqi Hong, Feng Ye

**Affiliations:** 1 Department of Medical Oncology, Xiamen Key Laboratory of Antitumor Drug Transformation Research, The First Affiliated Hospital of Xiamen University, School of clinical Medicine,, Fujian Medical University, No. 55 Zhenhai Road, Siming District, Xiamen, Fujian China; 2 Department of Gastroenterology, Xiangya Hospital of Centre-South University, Changsha, Hunan China

**Keywords:** KLF15, TFAP2A-AS1, NISCH, Gastric cancer (GC), Transcription factor

## Abstract

**Background:**

Recently, overwhelming evidence supports that long noncoding RNAs (lncRNAs) play crucial roles in the occurrence and progression of tumors. However, the role and mechanism of lncRNA TFAP2A-AS1 in human gastric cancer (GC) remains unclear. Thus, the biological role and regulatory mechanisms of TFAP2A-AS1 in GC were explored.

**Methods:**

Quantitative real-time PCR (qPCR) was applied to detect gene expression. Western blot was used to measure protein expression. Cell proliferation and migration were determined by functional assays. Fluorescence in situ hybridization (FISH) assays were performed to determine the subcellular distribution of TFAP2A-AS1 in GC. Mechanism investigations were conducted to explore the downstream genes of TFAP2A-AS1 and the upstream transcription factor of TFAP2A-AS1 in GC cells.

**Results:**

TFAP2A-AS1 inhibits the proliferation and migration of GC cells. In the downstream regulation mechanism, miR-3657 was verified as the downstream gene of TFAP2A-AS1 and NISCH as the target of miR-3657. NISCH also suppresses cell proliferation and migration in GC. In the upstream regulation mechanism, transcription factor KLF15 positively mediates TFAP2A-AS1 to suppress GC cell proliferation and migration.

**Conclusion:**

KLF15-mediated TFAP2A-AS1 hampers cell proliferation and migration in GC via miR-3657/NISCH axis.

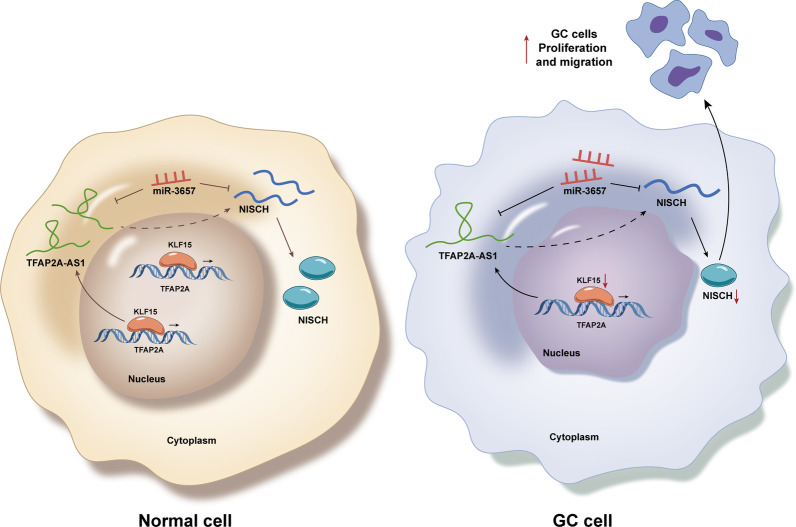

**Supplementary Information:**

The online version contains supplementary material available at 10.1186/s13062-021-00300-y.

## Background

Gastric cancer (GC) is one of the leading causes of cancer-related deaths around the world. The American Cancer Society statistics estimates that in 2021, approximately 26,560 cases will be diagnosed with stomach cancer and about 11,180 death cases in the United States. GC occurrence is multifactorial, many factors can exert a function in its incidence, including diet and dietary habits, smoking, alcoholic consumption, genetic factors, family history, infections, demographic characteristics, occupational exposure, ionizing radiation, and others [1]. In recent years, great advancements in the treatment options of advanced GC have been achieved, including neoadjuvant chemotherapy, radiation therapy, perioperative radiotherapy, immunotherapy, cell-cycle inhibitors, MMP inhibitors and molecular-targeted therapies, etc. [2]. At the same time, increasing evidence supports that molecular biomarkers contribute to the progress of prognostic prediction and early diagnosis for GC patients [3, 4]. Therefore, it is of great significance to explore promising biomarkers for developing effective treatment strategies for GC treatment.

LncRNAs, a group of non-coding RNAs (ncRNAs) are generally classified as over 200 nt long transcripts, lacking the coding sequence (CDS) or open reading frame (ORF) [5]. Previous studies have reported the functions of lncRNAs in the occurrence and progression of GC from the aspect of cell proliferation, migration and invasion, etc. For instance, HOXC-AS3 transcriptionally regulates the oncogenesis of GC [6]; SNHG1 contributes to GC cell proliferation [7]. Moreover, some have further investigated that the regulation mechanism of lncRNAs underlying in GC, such as ceRNA (competing endogenous RNA). For example, SNHG5/miR-32 axis mediates cell proliferation and migration via targeting KLF4 in GC [8]. TFAP2A-AS1 is a novel lncRNA which is seldom investigated. So far, TFAP2A-AS1 has been reported to be key prognostic lncRNA in clear cell renal cell carcinoma [9] and to inhibit cell proliferation and invasion in breast cancer via miR-933/SMAD2 axis [10]. However, the biological role and underlying mechanism of TFAP2A-AS1 in GC remains to be explored.

The current study aimed to investigate the role and upstream/downstream mechanism of TFAP2A-AS1 in the proliferation and migration of GC cells, which might provide new insights into GC treatment.

## Results

### TFAP2A-AS1 suppresses GC cell proliferation and migration

**Fig. 1:**
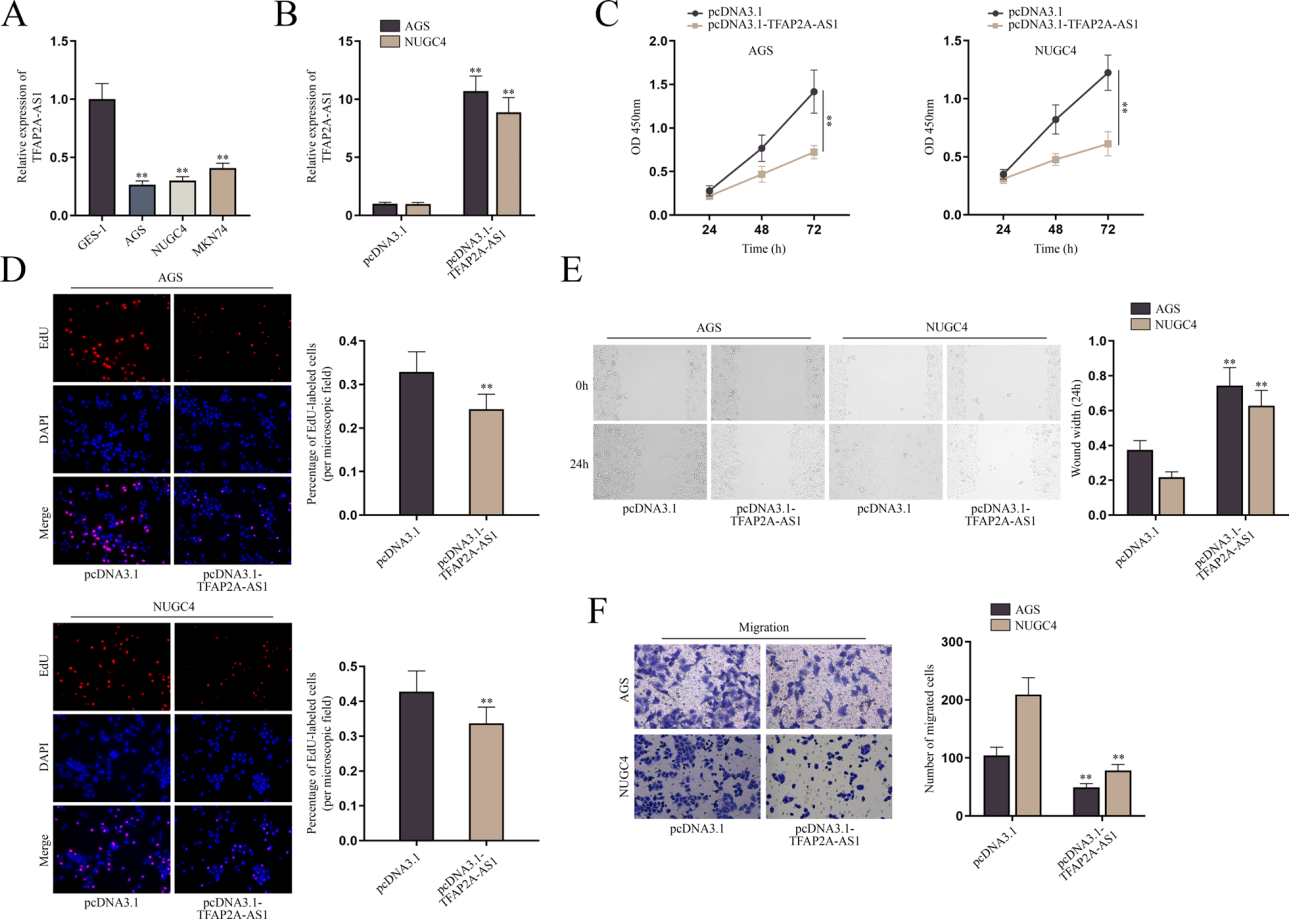
TFAP2A-AS1 suppresses GC cell proliferation and migration. **A** The expression of TFAP2A-AS1 in GES-1, AGS, NUGC4 and MKN74 cells was detected by qPCR. **B** The overexpression efficiency of pcDNA3.1-TFAP2A-AS1 was detected by qPCR. **C**–**F** CCK-8, EdU, wound healing and transwell assays were used to evaluate the proliferation and migration of AGS and NUGC4 cells after the overexpression of TFAP2A-AS1. The statistical analysis for **A** was one-way ANOVA, for **B**, **D**, **E**, **F** was student’s t-test, and for **C** was two-way ANOVA. β-actin was used as the internal reference for gene expression analysis. **P < 0.01

Firstly, we utilized starBase (http://starbase.sysu.edu.cn/), finding that TFAP2A-AS1 was upregulated in GC tissues compared with normal tissues (Additional file 1: Fig. S1A). QPCR was implemented to verify whether TFAP2A-AS1 was unusually expressed in AGS, NUGC4, and MKN74 cells, the common cells for GC researches [11, 12], compared with in GES-1 cells. It was clearly shown that TFAP2A-AS1 was lower expressed in GC cells lines, especially in AGS and NUGC4 cells, in comparison with GES-1 cells (Fig. 1A and Additional file 1: Fig. S1B). Thereby, AGS and NUGC4 cells were selected for the follow-up assay in this study. First, the overexpression efficiency of pcDNA3.1-TFAP2A-AS1 was testified through qPCR, and the result showed that TFAP2A-AS1 expression was distinctly elevated after the transfection of pcDNA3.1-TFAP2A-AS1 (Fig. 1B and Additional file 1: Fig. S1C). Then, CCK-8 assay showed the OD value at 450 nm was lower at every time point after the upregulation of TFAP2A-AS1 than the control group, suggesting the suppressed cell proliferation (Fig. 1C). Consistently, EdU assay revealed that the percentage of EdU-labeled cells was declined after the overexpression of TFAP2A-AS1 when compared with the control group, suggesting the inhibited proliferation aroused by TFAP2A-AS1 upregulation (Fig. 1D). Then, wound healing and transwell assays were applied for evaluating the migratory capacity of GC cells. Result clearly showed the wound width at the 24 h was significantly wider in pcDNA3.1-TFAP2A-AS1 group than in pcDNA3.1 group (Fig. 1E). Moreover, the number of migrated cells was obviously reduced after the transfection of pcDNA3.1-TFAP2A-AS1 in comparison with the control group (Fig. 1F). Therefore, the impaired migration of GC cells was observed. Taken together, TFAP2A-AS1 suppresses the proliferation and migration of GC cells.

### TFAP2A-AS1 sponges miR-3657 in GC cells

**Fig. 2:**
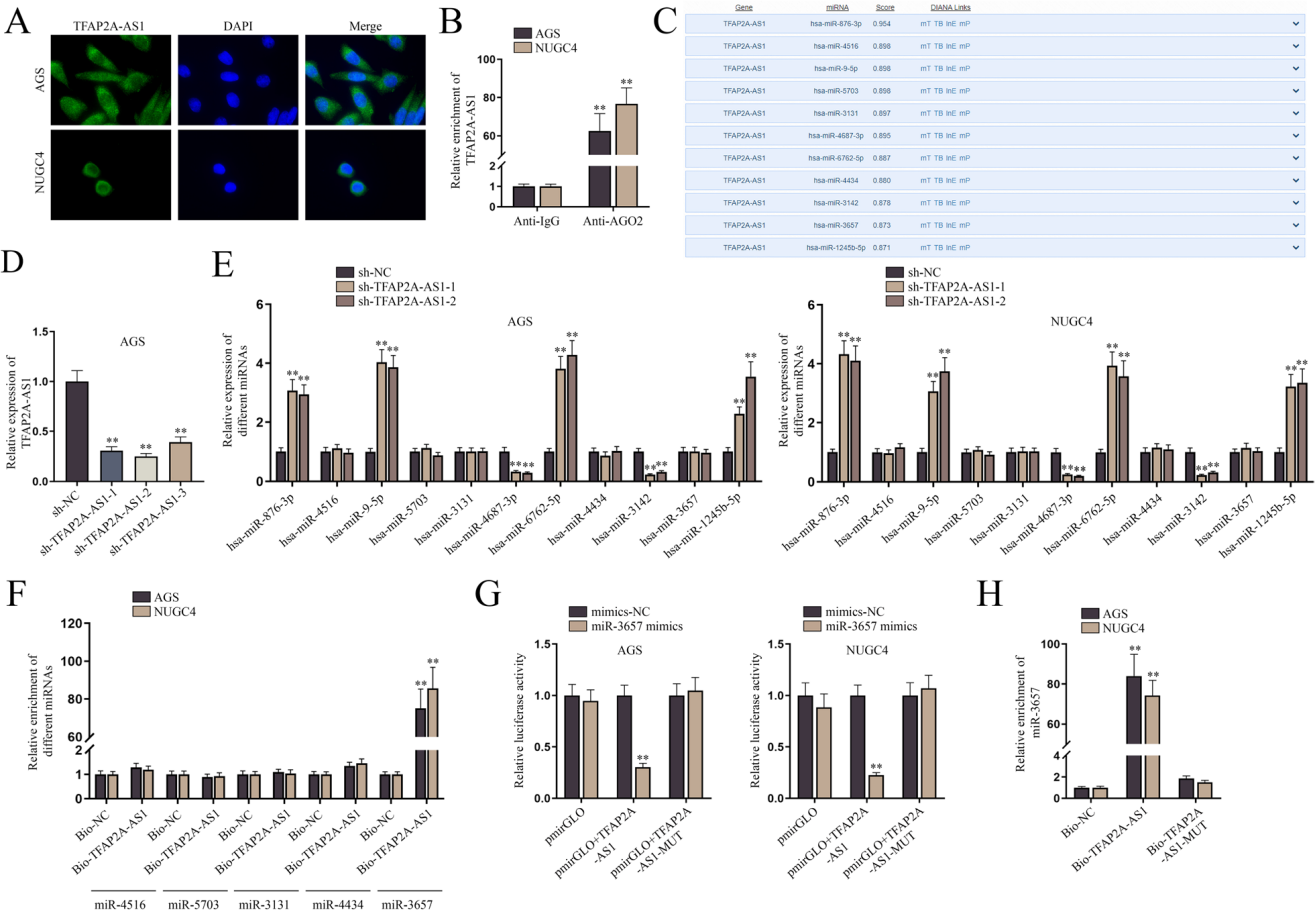
TFAP2A-AS1 sponges miR-3657 in GC cells. **A** FISH assay was used to detect the distribution of TFAP2A-AS1 in AGS and NUGC4 cells. **B** RIP assay was used to detect the enrichment of TFAP2A-AS1 in RISC of AGS and NUGC4 cells. **C** DIANA database was used to screen out the potential target miRNAs, hsa-miR-876-3p, hsa-miR-4516, hsa-miR-9-5p, hsa-miR-5703, hsa-miR-3131, hsa-miR-4687-3p, hsa-miR-6762-5p, hsa-miR-4434, hsa-miR-3142, hsa-miR-3657 and hsa-miR-1245b-5p. **D** QPCR was used to detect the knockdown efficiency of sh-TFAP2A-AS1-1/2/3 in AGS cells. **E** QPCR was used to detect the expression of potential target miRNAs in AGS and NUGC4 cells after the knockdown of TFAP2A-AS1. **F** RNA-pulldown assay was used to detect the interaction of TFAP2A-AS1 with hsa-miR-4516, hsa-miR-5703, hsa-miR-3131, hsa-miR-4434 or hsa-miR-3657 in AGS and NUGC4 cells. **G**, **H** Dual luciferase reporter and RNA-pulldown assays were used to explore the interaction between TFAP2A-AS1 and miR-3657 in AGS and NUGC4 cells. The statistical analysis for **B**, **E** was student’s t-test, for **D**, **G** was one-way ANOVA, and for **F** was two-way ANOVA. β-actin was used as the internal reference for gene expression analysis. **P < 0.01

CeRNA is one of the mechanisms through which lncRNAs functions in human cancer and cancer cells [10]. Hence, we wondered whether TFAP2A-AS1 worked as the upstream lncRNA and sponged certain miRNAs in GC cells. Given that the location of lncRNAs was critical for their functions, FISH assay was conducted to locate TFAP2A-AS1 in GC cells. It turned out that TFAP2A-AS1 was distributed mainly in the cytoplasm although it was located in both nuclei and the cytoplasm in GC cells (Fig. 2A). AGO2 is one of the essential elements of RNA-induced silencing complexes (RISCs) [13]. Hence, AGO2-RIP assay was performed to determine the participation of TFAP2A-AS1 in ceRNA mechanism. The result showed that TFAP2A-AS1 was prominently abundant in the Anti-AGO2 group while being little enriched in Anti-IgG precipitates, suggesting the existence of TFAP2A-AS1 in RISCs (Fig. 2B and Additional file 2: Fig. S2A). To figure out the downstream miRNAs of TFAP2A-AS1, DIANA (http://diana.imis.athena-innovation.gr/DianaTools/index.php; Score ≥ 0.87) was used to predict the miRNA candidates and 11 miRNAs (hsa-miR-876-3p, hsa-miR-4516, hsa-miR-9-5p, hsa-miR-5703, hsa-miR-3131, hsa-miR-4687-3p, hsa-miR-6762-5p, hsa-miR-4434, hsa-miR-3142, hsa-miR-3657 and hsa-miR-1245b-5p) were screened out (Fig. 2C). QPCR was used to detect the knockdown efficiency of sh-TFAP2A-AS1-1/2/3 in AGS cells (Fig. 2D and Additional file 2: Fig. S2B). Next, qPCR was used to measure the expressions of these 11 miRNAs before and after the knockdown of TFAP2A-AS1 in GC cells. The expression of hsa-miR-4516, hsa-miR-5703, hsa-miR-3131, hsa-miR-4434 and hsa-miR-3657 was not significantly altered by the downregulation of TFAP2A-AS1 whereas the other miRNAs were distinctly upregulated or downregulated by the transfection of sh-TFAP2A-AS1-1/2 plasmids (Fig. 2E and Additional file 2: Fig. S2C). Therefore, the five miRNAs were selected in the following RNA pulldown assays to determine the bona fide miRNA sponged by TFAP2A-AS1 in GC cells. It was manifested that miR-3657 was much more precipitated by Bio-TFAP2A-AS1 than the other miRNAs, indicating miR-3657 was the downstream target of TFAP2A-AS1 (Fig. 2F and Additional file 2: Fig. S2D). To further investigate the interaction between TFAP2A-AS1 and miR-3657, luciferase reporter assays were conducted, showing that the transfection of miR-3657 mimics overtly impaired the luciferase activity of pmirGLO + TFAP2A-AS1 while barely influencing that of pmirGLO or pmirGLO + TFAP2A-AS1-MUT in GC cells (Fig. 2G). Moreover, the pulldown assay uncovered that miR-3657 was significantly enriched in Bio-TFAP2A-AS1 precipitates but seldom in the Bio-NC or Bio-TFAP2A-AS1-MUT pull-downs, validating the combination between TFAP2A-AS1 and miR-3657 (Fig. 2H and Additional file 2: Fig. S2E). In conclusion, TFAP2A-AS1 sponges miR-3657 in GC cells.

### NISCH is the downstream mRNA of miR-3657

**Fig. 3:**
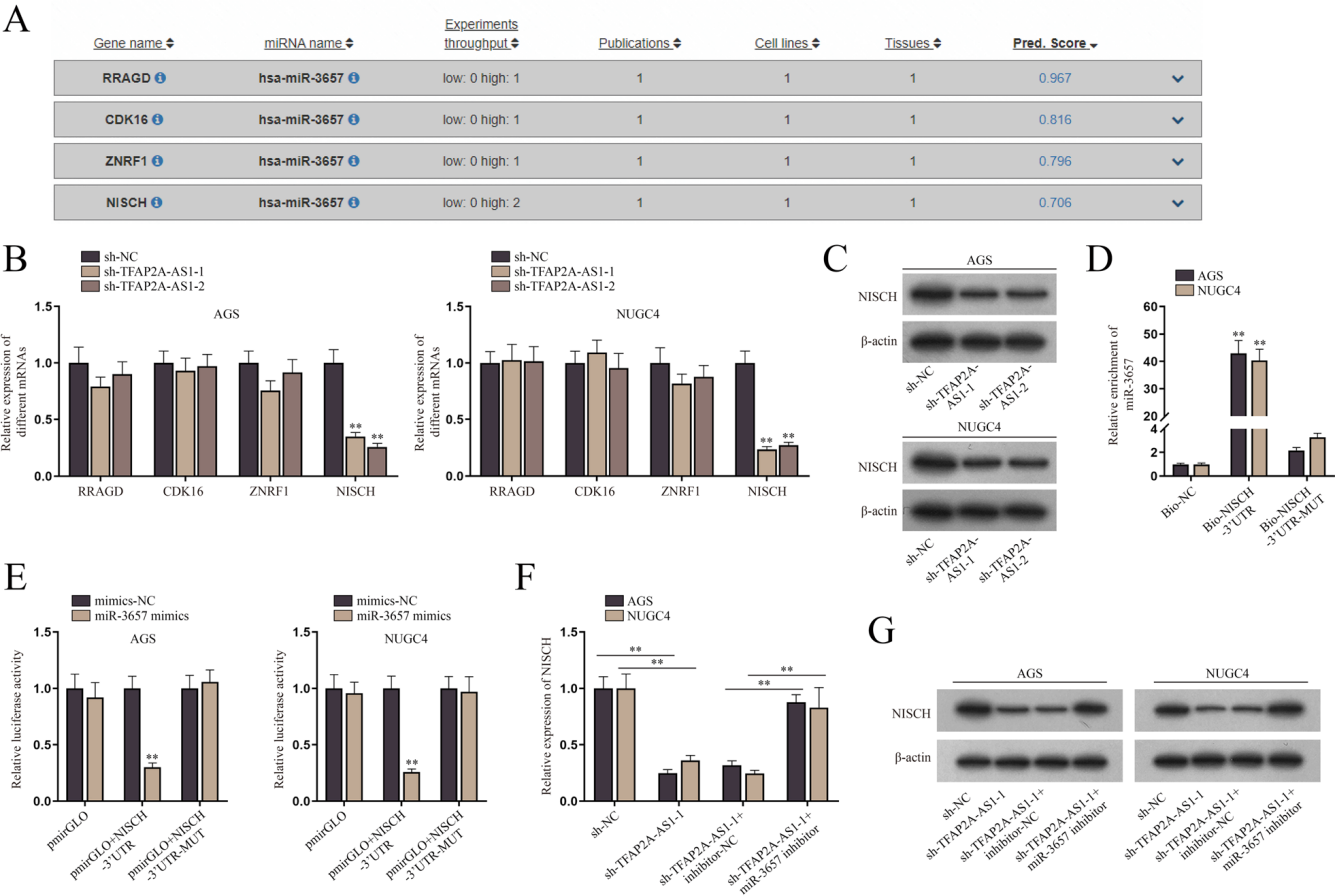
NISCH is the downstream mRNA of miR-3657. **A** DIANA database was utilized to screen out the potential target mRNAs, RRAGD, CDK16, ZNRF1 and NISCH. **B** QPCR was used to evaluate the expression of potential target mRNAs after the knockdown of TFAP2A-AS1 in AGS and NUGC4 cells. **C** Western blot analysis was used to assess the protein level of NISCH in AGS and NUGC4 cells after the knockdown of TFAP2A-AS1. **D**, **E** RNA-pulldown and dual luciferase reporter assays were used to verify the interaction between NISCH and miR-3657 in AGS and NUGC4 cells. **F**, **G** QPCR and western blot analyses was used to detect the level of NISCH in AGS and NUGC4 cells after the transfection of sh-NC, sh-TFAP2A-AS1-1, sh-TFAP2A-AS1-1 + inhibitor-NC or sh-TFAP2A-AS1-1 + miR-3657 inhibitor. The statistical analysis for **B**, **D**, **F** was one-way ANOVA, and for **E** was two-way ANOVA. β-actin was used as the internal reference for gene expression analysis. **P < 0.01

Via the utilization of DIANA database, we screened out the potential target mRNAs of miR-3657 namely, RRAGD, CDK16, ZNRF1 and NISCH as shown in Fig. 3A (score ≥ 0.70). Then qPCR was conducted to detect the expression of all the candidates in AGS and NUGC4 cells after the knockdown of TFAP2A-AS1. The results showed that only the expression of NISCH was significantly reduced after TFAP2A-AS1 inhibition (Fig. 3B and Additional file 3: Fig. S3A). Then we conducted western blot analysis, proving the correlation between TFAP2A-AS1 and NISCH at protein level (Fig. 3C and Additional file 3: Fig. S3B). Therefore, we selected NISCH for the follow-up experiments. After the implementation of RNA-pulldown assay, qPCR was performed to measure the enrichment of miR-3657 in AGS and NUGC4 cells. The results showed that miR-3657 was preferentially enriched in Bio-NISCH-3′ UTR group, verifying its binding to the 3′UTR of NISCH (Fig. 3D and Additional file 3: Fig. S3C). Next, the interaction between NISCH 3′UTR and miR-3657 was further proved by dual luciferase reporter assay, as the luciferase activity of pmirGLO + NISCH-3′UTR group was remarkably reduced after the transfection of miR-3657 mimics (Fig. 3E). Subsequently, rescue experiments were conducted to explore the relationship among TFAP2A-AS1, miR-3657 and NISCH. The mRNA and protein levels of NISCH were evaluated by qPCR and western blot analyses after the transfection of sh-NC, sh-TFAP2A-AS1-1, sh-TFAP2A-AS1-1 + inhibitor-NC or sh-TFAP2A-AS1-1 + miR-3657 inhibitor. The results showed that, miR-3657 depletion reversed NISCH silencing by TFAP2A-AS1 ablation at both mRNA and protein levels, suggesting that TFAP2A-AS1 upregulated NISCH expression via competitively binding to miR-3657 (Fig. 3F, G and Additional file 3: Fig. S3D, E). Taken together, NISCH is the downstream mRNA of miR-3657.

### NISCH inhibits the proliferation and migration of GC cells

**Fig. 4:**
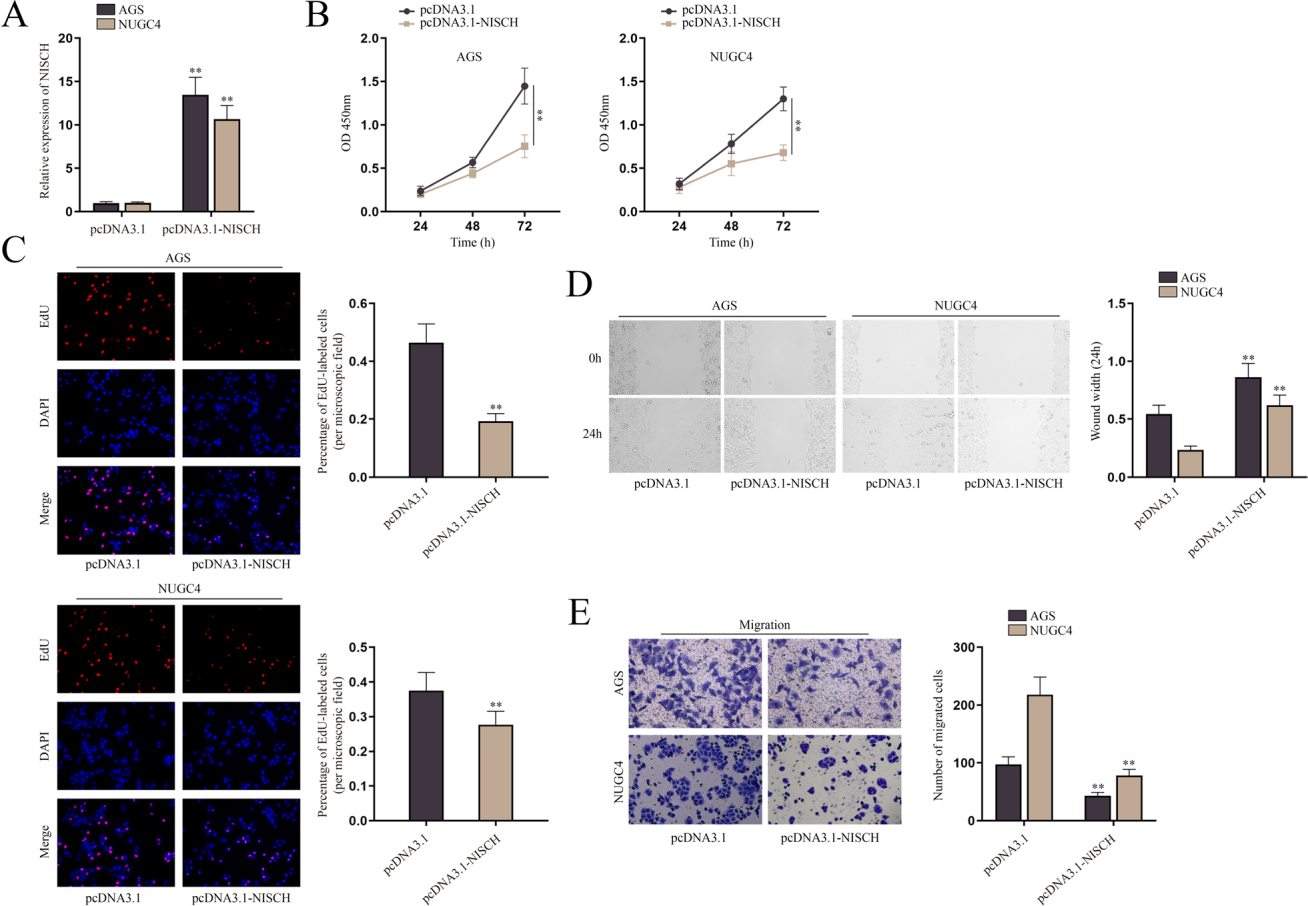
NISCH inhibits the proliferation and migration of GC cells. **A** QPCR was performed to detect the overexpression efficiency of pcDNA3.1-NISCH in AGS and NUGC4 cells. **B**–**E** CCK-8, EdU, wound healing and transwell assays were used to evaluate the proliferation and migration of AGS and NUGC4 cells after the overexpression of NISCH. The statistical analysis for **A**, **C**, **D**, **E** was student’s t-test, and for **B** was two-way ANOVA. β-actin was used as the internal reference for gene expression analysis. **P < 0.01

Next, we investigated the effect of NISCH on cell proliferation and migration of GC. Firstly, we assessed the overexpression efficiency of pcDNA3.1-NISCH (Fig. 4A and Additional file 4: Fig. S4A). Afterwards, a series of functional experiments were implemented to research the biological functions of NISCH in GC cells. The results of CCK-8 and EdU assays showed that NISCH overexpression suppressed the proliferation of AGS and NUGC4 cells, as OD value and the number of EdU-positive cells was both reduced in pcDNA3.1-NISCH groups (Fig. 4B, C). Furthermore, the results of wound healing and transwell assays showed that the migration of AGS and NUGC4 cells was inhibited after the transfection of pcDNA3.1-NISCH (Fig. 4D, E). To sum up, NISCH inhibits the proliferation and migration of GC cells.

### TFAP2A-AS1 is positively regulated by transcription factor KLF15

Previously, we found that TFAP2A-AS1 could inhibit the proliferation and migration of GC cells by competitively sponging miR-3657 to upregulate NISCH expression, but the regulatory mechanism of TFAP2A-AS1 was still unclear. It has been reported that transcription factors play important roles in cancer [14]. However, the transcription factors regulating TFAP2A-AS1 have not been reported before. Hence, we next explored the transcription factors that might regulate TFAP2A-AS1.

**Fig. 5:**
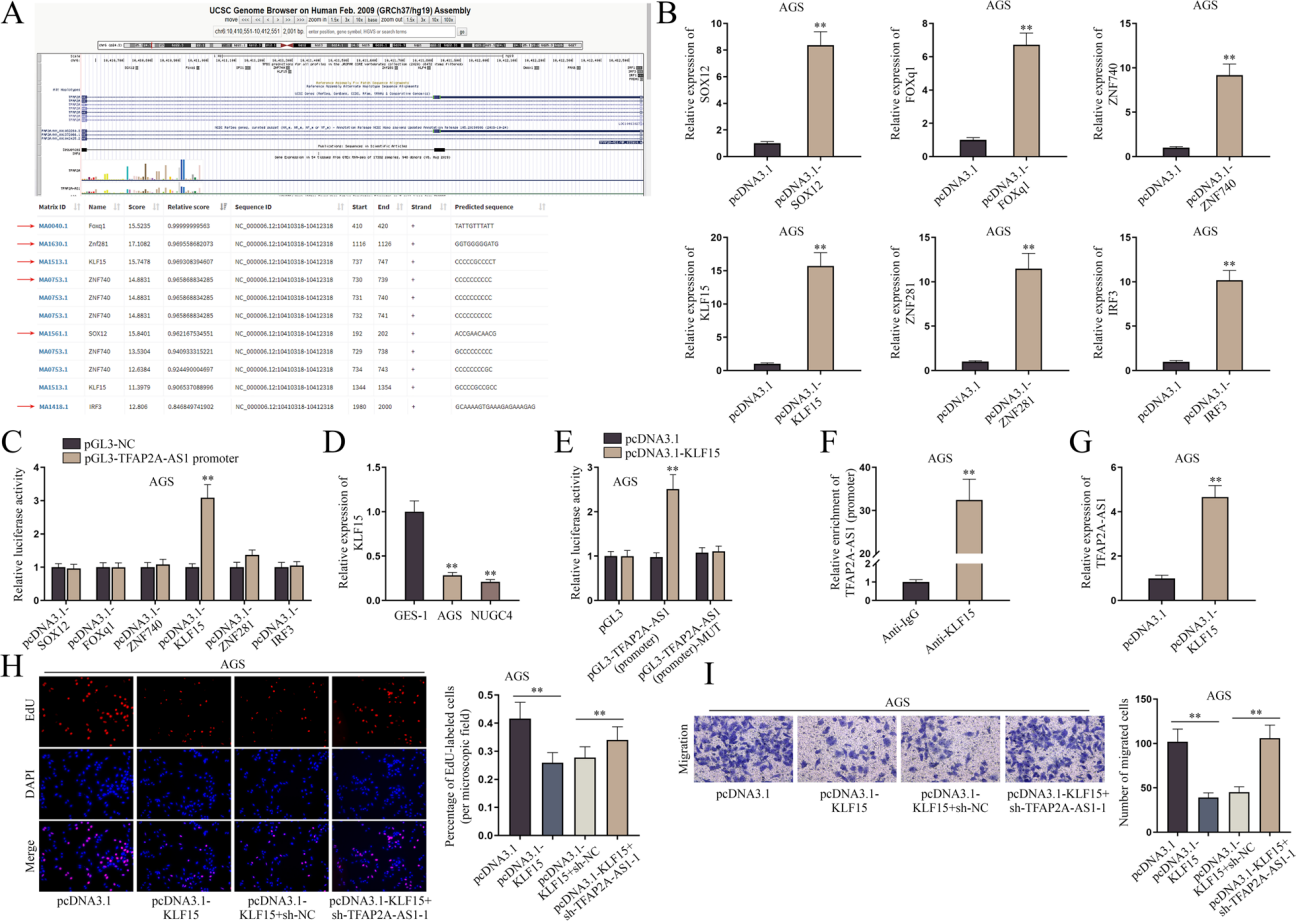
TFAP2A-AS1 is positively regulated by transcription factor KLF15. **A** UCSC and JASPAR were used to predict the potential mRNA, SOX12, FOXq1, ZNF740, KLF15, ZNF281 and IRF3, and their binding sites of TFAP2A-AS1 promoter. **B** QPCR was used to evaluate the overexpression efficiency of pcDNA3.1-SOX12, pcDNA3.1-FOXq1, pcDNA3.1-ZNF740, pcDNA3.1-KLF15, pcDNA3.1-ZNF281 and pcDNA3.1-IRF3 in AGS cells. **C** Dual luciferase reporter assay was conducted to verify the interaction between TFAP2A-AS1 promoter and all the candidates in AGS cells. **D** QPCR was conducted to evaluate the expression of KLF15 in AGS, NUGC4 and GES-1 cells. **E**, **F** Dual luciferase reporter and ChIP assays verified the interaction between TFAP2A-AS1 promoter and KLF15 in AGS cells. **G** QPCR detected the expression of TFAP2A-AS1 after the overexpression of KLF15 in AGS cells. **H**, **I** EdU and transwell assays were implemented in AGS cells after the transfection of pcDNA3.1, pcDNA3.1-KLF15, pcDNA3.1-KLF15 + sh-NC or pcDNA3.1-KLF15 + sh-TFAP2A-AS1-1. The statistical analysis for **B**, **F**, **G** was student’s t-test, for **C** was two-way ANOVA, and for **D**, **E**, **H**, **I**, **J** was one-way ANOVA. β-actin was used as the internal reference for gene expression analysis. **P < 0.01

After linking the website UCSC (https://genome.ucsc.edu/) to the data of the other website, JASPAR (http://jaspar.genereg.net/), we then utilized it to predict potential transcription factors of TFAP2A-AS1 with prediction score set at 550. After the removal of genes with reversed order of expression, six candidates were screened out, SOX12, FOXq1, ZNF740, KLF15, ZNF281 and IRF3. Then the binding sites of these six candidates to TFAP2A-AS1 promoter were predicted by JASPAR (Fig. 5A). Afterwards, qPCR was conducted to detect the overexpression efficiency of pcDNA3.1-SOX12, pcDNA3.1-FOXq1, pcDNA3.1-ZNF740, pcDNA3.1-KLF15, pcDNA3.1-ZNF281 and pcDNA3.1-IRF3 in AGS cells (Fig. 5B and Additional file 4: Fig. S4B). Subsequently, dual luciferase reporter assay was performed to screen the transcription factors capable of binding to the TFAP2A-AS1 promoter. The results indicated the interaction between KLF15 and TFAP2A-AS1 promoter, as evidenced by the obvious increase of luciferase activity (Fig. 5C). We then used qPCR to detect the expression of KLF15, discovering that KLF15 was downregulated in AGS and NUGC4 cells and was upregulated in GES-1 cells (Fig. 5D and Additional file 4: Fig. S4C). Next, we performed dual luciferase reporter and ChIP assays in AGS cells, further proving the interaction between KLF15 and TFAP2A-AS1 promoter (Fig. 5E, F and Additional file 4: Fig. S4D). Then, qPCR was performed to detect the expression of TFAP2A-AS1 after the overexpression of KLF15. The results showed that the expression of TFAP2A-AS1 was upregulated after the overexpression of KLF15, indicating that KLF15 positively regulated TFAP2A-AS1 (Fig. 5G and Additional file 4: Fig. S4E). Rescue experiments were conducted to figure out the underlying mechanism between KLF15, TFAP2A-AS1 and biological behaviors of GC cells. We performed EdU and transwell assays in AGS cells after the transfection of pcDNA3.1, pcDNA3.1-KLF15, pcDNA3.1-KLF15 + sh-NC or pcDNA3.1-KLF15 + sh-TFAP2A-AS1-1. The results showed that TFAP2A-AS1 interference reversed the inhibition of proliferation and migration by KLF15 overexpression (Fig. 5H, I). Taken together, transcription factor KLF15 inhibits cell proliferation and migration of GC by positively regulating TFAP2A-AS1 expression.

## Discussion

As a neoplastic disease, GC is one of the leading causes of malignancy-related death [15]. GC could be detected in the early stage to be diagnosed. CEA, DNA methylation, CA19-9, CA125, PG and lncRNA have been believed to be the common biomarkers for the diagnosis of early GC [16]. However, the detection of early GC remains a challenge for the lack of effective diagnostic markers [17]. Hence, the exploration of more biomarkers for GC diagnosis and treatment is critically needed.

In this study, TFAP2A-AS1 was found to be unusually downregulated in GC cell lines, inhibiting the proliferation and migration of GC cells. Previously, TFAP2A-AS1 was scarcely researched and its functional influence on human cancer cells is also seldom found. According to the existing literatures, the suppressive function of TFAP2A-AS1 in breast cancer has been ascertained [18]. Furthermore, TFAP2A-AS1 has been proved to be relative to the prognosis of clear cell renal cell carcinoma [9]. Nonetheless, the diagnostic or prognostic potential of TFAP2A-AS1 has not been investigated in GC. Thereby, this current study is supposed to make a supplement to the research on TFAP2A-AS1 in GC cells to provide a novel insight for the exploration of GC. The study on miR-3657 is also limited, but its influence on GC cell apoptosis has been proved. It was determined that circRACGAP1 sponges miR-3657 to accelerate the autophagy of GC cells to reduce the sensitivity of GC cells to apatinib [19]. In this study, we have found another ceRNA mechanism of miR-3657 that TFAP2A-AS1 sponges miR-3657 to impair GC cell proliferation and migration. Thereby, this study provides a novel mechanism of miR-3657 in GC cells and offers one more potential target axis for GC treatment. Different from TFAP2A-AS1 and miR-3657, NISCH has been more reported in the field of functions in human cancers. To be listed, NISCH was found to be aberrantly downregulated in ovarian cancer cells, inhibiting the proliferation and invasion of tumor cells through suppressing FAK signaling pathway [20]. Moreover, it was verified that NISCH attenuates the apoptosis of PC12 cells (neurocyte) [21]. The tumor and disease suppressor role of NISCH has also been identified in lung cancer [22]. Consistent with previous studies, this study has discovered that NISCH is positively regulated by TFAP2A-AS1 sponging miR-3657 to impede GC cell proliferation and migration. Hence, this study is the first discovery which not only relates NISCH to GC cells but also to TFAP2A-AS1. Besides the ceRNA mechanism of TFAP2A-AS1, the transcription factor of TFAP2A-AS1 has been explored, too. In this study, KLF15 was determined as the upstream transcriptionally regulating the expression of TFAP2A-AS1 through ChIP, qPCR and a series of rescue assays. According to precious literatures, KLF15 has been proved promoting the transcription of KLF3 in bovine adipocytes [23] and the ectopic expression of KLF15 was discovered to affect the rhodopsin condition [24]. Therefore, KLF15 is a critical factor in mammals. In addition, KLF15 could propel lung adenocarcinoma cell proliferation and metastasis [25]. In contrast, KLF15 upregulates the expression of CDKN1A/p21 and CDKN1C/p57 to impair the proliferation of GC cells [26]. Thereby, the discovery of the upregulating modulation of TFAP2A-AS1 in this study is another evidence of the inhibitory role of KLF15 in human cancers.

In conclusion, this study has demonstrated the suppressive function of KLF15/TFAP2A-AS1/miR-3657/NISCH axis in GC cells, which offers a novel potential mechanism for being targeted in the treatment of GC. However, in vivo assays were in lack and the pathological features need to be taken into consideration to make the result in this study supported by the clinical data. Moreover, the functional assays after the alteration of miR-3657 alone need to be performed to validate its biological influence on GC cells.

## Materials and methods

### Cell lines and cultivation

GC cell lines (AGS, NUGC4, and MKN74) and human normal epithelial cell line (GES-1) were purchased from the American Type Culture Collection (ATCC; Manassas, VA, USA). Cells were cultured in the RPMI1640 medium (CD-02168-ML, GIBCO, USA) added with 10% Fetal Bovine Serum (FBS; 10270-106, GIBCO) and 100 U/mL Penicillin/Streptomycin solution in humidified incubators. The air and temperature for the cell cultivation was required of 5% CO_2_ and 37 °C respectively.

### Vector construction and cell transfection

For the overexpression of TFAP2A-AS1, NISCH, SOX12, FOXq1, ZNF740, KLF15, ZNF281 and IRF3, the full length of these of these RNAs were inserted into pcDNA3.1 vectors to construct pcDNA3.1-TFAP2A-AS1, pcDNA3.1-NISCH, pcDNA3.1-SOX12, pcDNA3.1-FOXq1, pcDNA3.1-ZNF740, pcDNA3.1-KLF15, pcDNA3.1-ZNF281, and pcDNA3.1-IRF3 plasmids. For the knockdown of TFAP2A-AS1, sh-TFAP2A-AS1-1/2/3 plasmids were chemically synthesized by RiboBio (China). For the overexpression or silence of miR-3657, miR-3657 mimics or inhibitor were synthesized with the sequence or complementary bases of miR-3657. For the luciferase report assay, the wild-type or mutant TFAP2A-AS1 or NISCH-3′UTR was sub-clone into pmirGLO to form pmirGLO + TFAP2A-AS1/pmirGLO + TFAP2A-AS1-MUT or pmirGLO + NISCH-3′UTR (3′ untranslated region)/pmirGLO + NISCH-3′UTR-MUT, TFAP2A-AS1 promoter or TFAP2A-AS1 promoter-MUT being sub-cloned into pGL3 vectors to construct pGL3-TFAP2A-AS1 promoter or pGL3-TFAP2A-AS1 promoter-MUT. All the transfections were conducted with the application of Lipofectamine 2000 (XFSJ16444, GIBCO).

### QPCR

After being extracted from GC cells via Trizol (abs60154, Absin, Shanghai, China), total RNAs were reversely transcribed into complementary DNAs (cDNAs) using 1st Strand cDNA Synthesis Kit (11141ES10, Takara, Japan). Then, qRT-PCR Kit (QR0100-1KT, Sigma-Aldrich, USA) was used for PCR and evaluation of the relative expression levels of different RNAs: TFAP2A-AS1, RRAGD, CDK16, ZNRF1, NISCH, SOX12, FOXq1, ZNF740, KLF15, ZNF281, IRF3, hsa-miR-876-3p, hsa-miR-4516, hsa-miR-9-5p, hsa-miR-5703, hsa-miR-3131, hsa-miR-4687-3p, hsa-miR-6762-5p, hsa-miR-4434, hsa-miR-3142, hsa-miR-3657 and hsa-miR-1245b-5p. The results were calculated based on 2^−ΔΔCt^ method. β-actin and GAPDH served as the internal reference for this analysis.

### Western blot

For the extraction of total proteins from GC cell, ProteoPrep^®^ Total Extraction Sample Kit (PROTTOT-1KT, Sigma-Aldrich, USA) was implemented in this assay. Then, proteins were subjected to electrophoresis in SDS-PAGE Kit (P1200, Solarbio, China) before being transferred to PVDF membranes. Next, membranes were blocked with 5% skim milk at room temperature for 1 h, followed by the incubation with Anti-GAPDH (ab8245, Abcam, UK), Anti-NISCH (ab240588, Abcam, UK) or Anti-β-actin (ab8226, Abcam) at 4 °C for the whole night. After the subsequent cultivation of membranes with secondary antibodies, the protein levels were analyzed with the application of ECL substrates. β-actin and GAPDH served as the internal reference for this analysis. The results were quantified using ImageJ.

### Dual-luciferase reporter assay

For the luciferase reporter assay assessing the interaction among RNAs, miR-3657 NC and miR-3657 mimics were co-transfected into 1 × 10^4^ GC cells with different luciferase vectors (pmirGLO/pmirGLO + TFAP2A-AS1/pmirGLO + TFAP2A-AS1-MUT or pmirGLO + NISCH-3′UTR/pmirGLO + NISCH-3′UTR-MUT). For the verification of transcription of TFAP2A-AS1, pcDNA3.1 or pcDNA3.1-KLF15/pcDNA3.1-SOX12/pcDNA3.1-FOXq1/pcDNA3.1-ZNF740/pcDNA3.1-ZNF281/pcDNA3.1-IRF3 was co-transfected with pGL3, pGL3-TFAP2A-AS1 promoter or pGL3-TFAP2A-AS1 promoter-MUT into GC cells. The luciferase activity was observed through the XSP-63B fluorescence microscopy (Shanghai Optical Instrument Factory, Shanghai, China).

### RNA pulldown assay

Structure buffer was added to biotinylated RNAs to form secondary structure. Afterwards, biotinylated RNAs were subjected to the heating and ice-bathing for denaturation. Denatured biotinylated (Bio-) TFAP2A-AS1, TFAP2A-AS1-MUT, NISCH-3′UTR or Bio-NISCH-3′UTR-MUT (1 µg) was incubated with 15µL Streptavidin beads for 2 h at 4 °C. Then, 2 × 10^7^ GC cells were lysed and the cell lysate was incubated with Bio-NC, Bio-TFAP2A-AS1, Bio-TFAP2A-AS1-MUT, Bio-NISCH-3′UTR, or Bio-NISCH-3′UTR-MUT conjugated with the beads at 4 °C overnight. After the precipitation was fulfilled, RNAs were extracted from precipitates for qPCR analysis.

### RNA Binding Protein Immunoprecipitation (RIP) assay

Imprint^®^ RNA Immunoprecipitation Kit (RIP-12RXN, Sigma-Aldrich, USA) was applied in this assay under the instruction of the manufacturer. First, Anti-IgG or Anti-Ago2 was cultured with 50 µg Protein A/G Agarose beads overnight at 4 °C. Meanwhile, 6 × 10^7^ GC cells were lysed in RIPA lysis buffer (RIPA20110527, TBD, China) into 300 µL lysate. The cell lysate was separated into three groups: Anti-IgG (100 µL lysate), Anti-Ago2 (100 µL) and Input (10 µL). After the incubation of antibodies overnight at 4 °C, the precipitates containing Protein A/G Agarose beads, Anti-IgG/Ago2 and RNAs were precipitated. QPCR analysis was implemented for the analysis of RNA enrichment. Anti-Ago2 (ab186733) was bought from Abcam.

### Chromatin immunoprecipitation (ChIP) assay

For this assay, 2 × 10^7^ GC cells were fixed with formaldehyde and subjected to the fractionation of nuclei and cytoplasm. Then, sonication was applied for the fragmentation of chromatins. Subsequently, Anti-IgG and Anti-KLF15 were incubated with chromatins for the immunoprecipitation. When the precipitation was finished, DNAs were extracted from the antibody-DNA complexes for qPCR.

### FISH assay

The FISH Kit (Bes1001, Biosense, China) was applied for FISH assays according the manufacturer’s protocol. 5 × 10^4^ GC cells were fixed with PFA and permeabilized with Triton X-100. Then, TFAP2A-AS1 probes labeled with Digoxigenin (DIG) were incubated with GC cells. DAPI was used for the counterstaining of nuclei. Finally, LSM800 confocal laser scanning microscopy (Carl Zeiss, Germany) was applied for the observation and recording of images.

### Cell Counting Kit-8 (CCK-8) assay

GC cells were plated into 96-well plate, with 1 × 10^3^ cells to each well, for cultivation with 5% CO_2_ at 37 ℃. After 24, 48 or 72 h, CCK-8 solution was added to the cells. The cell samples were incubated with CCK-8 solution for another 4 h. OD value at 450 nm was detected via a microplate reader to manifest the proliferation of GC cells.

### 5-ethynyl-2′-deoxyuridine (EdU) assay

After the transfection, 1 × 10^5^ GC cells were plated into 96-well plate for the cultivation overnight. Then, paraformaldehyde was used to fix cells and Triton X-100 to permeabilize cells. After that, EdU reagent was added to cells for staining. Nuclei were counterstained with DAPI. The cell viability was determined by a fluorescence microscope.

### Transwell assay

For the transwell-migration assay, 5 × 10^4^ transfected GC cells were re-suspended in the upper chambers without serum. Meanwhile, the lower chambers were filled with DMEM added with FBS. After the incubation for 24 h, cells maintained in the upper chambers were slightly swiped with a cotton swab and cells migrating to the lower chambers were fixed with methanol and stained with 0.1% crystal violet. Then, cells in the lower chambers were counted under the microscopy.

### Wound healing assay

5 × 10^5^ transfected GC cells were seeded into the 6-well plated for cultivation and a straight wound was scratched with a sterile pipette tip. After 24 h, the closure of wounds were recorded and measured under the microscopy at the magnification of 10 × 10. The wound width at the 24th h was normalized to that at 0 h. The results were quantified using ImageJ.

### Statistical analysis

All assays in this present study are required of triplicate and data were shown as mean ± standard deviation (SD) in each group. Statistical analysis was achieved with the application of SPSS 22.0 (IBM, Armonk, USA). For the statistical test, Student’s t test was used for the comparison between two groups and one-way or two-way analysis of variance (ANOVA) for that among more than two groups. The post hoc was achieved via Dunnett or Tukey. Data were significant only when the P value was less than 0.05.

## Supplementary Information

**Additional file 1: Fig. S1.** (A) StarBase database (http://starbase.sysu.edu.cn/) was used to detect the expression of TFAP2A-AS1 in GC tissues and normal tissues. (B) The expression of TFAP2A-AS1 in GES-1, AGS, NUGC4 and MKN74 cells was detected by qPCR. (C) The overexpression efficiency of pcDNA3.1-TFAP2A-AS1 was detected by qPCR. The statistical analysis for Figure S1A and S1C was t-test, and for Figure S1B was two-way ANOVA. GAPDH was used as the internal reference for gene expression analysis. **P < 0.01.

**Additional file 2: Fig. S2.** (A) RIP assay was used to detect the enrichment of TFAP2A-AS1 in RISC of AGS and NUGC4 cells. (B) QPCR was used to detect the knockdown efficiency of sh-TFAP2A-AS1-1/2/3 in AGS cells. (C) QPCR was used to detect the expression of potential target miRNAs in AGS and NUGC4 cells after the knockdown of TFAP2A-AS1. (D) RNA-pulldown assay was used to detect the interaction of TFAP2A-AS1 with hsa-miR-4516, hsa-miR-5703, hsa-miR-3131, hsa-miR-4434 or hsa-miR-3657 in AGS and NUGC4 cells. (E) RNA-pulldown assay was used to explore the interaction between TFAP2A-AS1 and miR-3657 in AGS and NUGC4 cells. The statistical analysis for Figure S2A was student’s t-test, and for Figure S2B, S2C, S2D and S2E was one-way ANOVA. GAPDH was used as the internal reference for gene expression analysis. **P < 0.01.

**Additional file 3: Fig. S3.** (A) QPCR was used to evaluate the expression of potential target mRNAs after the knockdown of TFAP2A-AS1 in AGS and NUGC4 cells. (B) Western blot analysis was used to assess the protein level of NISCH in AGS and NUGC4 cells after the knockdown of TFAP2A-AS1. (C) RNA-pulldown assay was used to verify the interaction between NISCH and miR-3657 in AGS and NUGC4 cells. (D-E) QPCR and western blot analyses was used to detect the level of NISCH in AGS and NUGC4 cells after the transfection of sh-NC, sh-TFAP2A-AS1-1, sh-TFAP2A-AS1-1+inhibitor-NC or sh-TFAP2A-AS1-1+miR-3657 inhibitor. The statistical analysis for Figure S3A, S3C and S3D was one-way ANOVA. GAPDH was used as the internal reference for gene expression analysis. **P < 0.01.

**Additional file 4: Fig. S4.** (A) QPCR was performed to detect the overexpression efficiency of pcDNA3.1-NISCH in AGS and NUGC4 cells. (B) QPCR was used to evaluate the overexpression efficiency of pcDNA3.1-SOX12, pcDNA3.1-FOXq1, pcDNA3.1-ZNF740, pcDNA3.1-KLF15, pcDNA3.1-ZNF281 and pcDNA3.1-IRF3 in AGS cells. (C) QPCR was conducted to evaluate the expression of KLF15 in AGS, NUGC4 and GES-1 cells. (D) ChIP assays verified the interaction between TFAP2A-AS1 promoter and KLF15 in AGS cells. (E) QPCR detected the expression of TFAP2A-AS1 after the overexpression of KLF15 in AGS cells. The statistical analysis for Figure S4A, S4B, S4D and S4E was student’s t-test, and for Figure S4C was one-way ANOVA. GAPDH was used as the internal reference for gene expression analysis. **P < 0.01.

## Data Availability

Not applicable.

## Abbreviations

GC: Gastric cancer
qPCR: Quantitative real-time PCR
FISH: Fluorescence in situ hybridization
ncRNAs: Non-coding RNAs
lncRNAs: Long noncoding RNAs
CDS: Coding sequence
ORF: Open reading frame
ceRNA: Competing endogenous RNA
FBS: Fetal bovine serum
ATCC: American Type Culture Collection
SD: Standard deviation
ANOVA: Analysis of variance
RISCs: RNA-induced silencing complexes
RIP: RNA Binding Protein Immunoprecipitation
ChIP: Chromatin immunoprecipitation
CCK-8: Cell Counting Kit-8
EdU: 5-Ethynyl-2′-deoxyuridine
