# Supplementary figures and images for: Transcription factor KLF15 inhibits the proliferation and migration of gastric cancer cells via regulating the TFAP2A-AS1/NISCH axis
Source: Biol Direct. 2021 Nov 3;16:21. doi: 10.1186/s13062-021-00300-y (PMC8565027; doi:10.1186/s13062-021-00300-y)

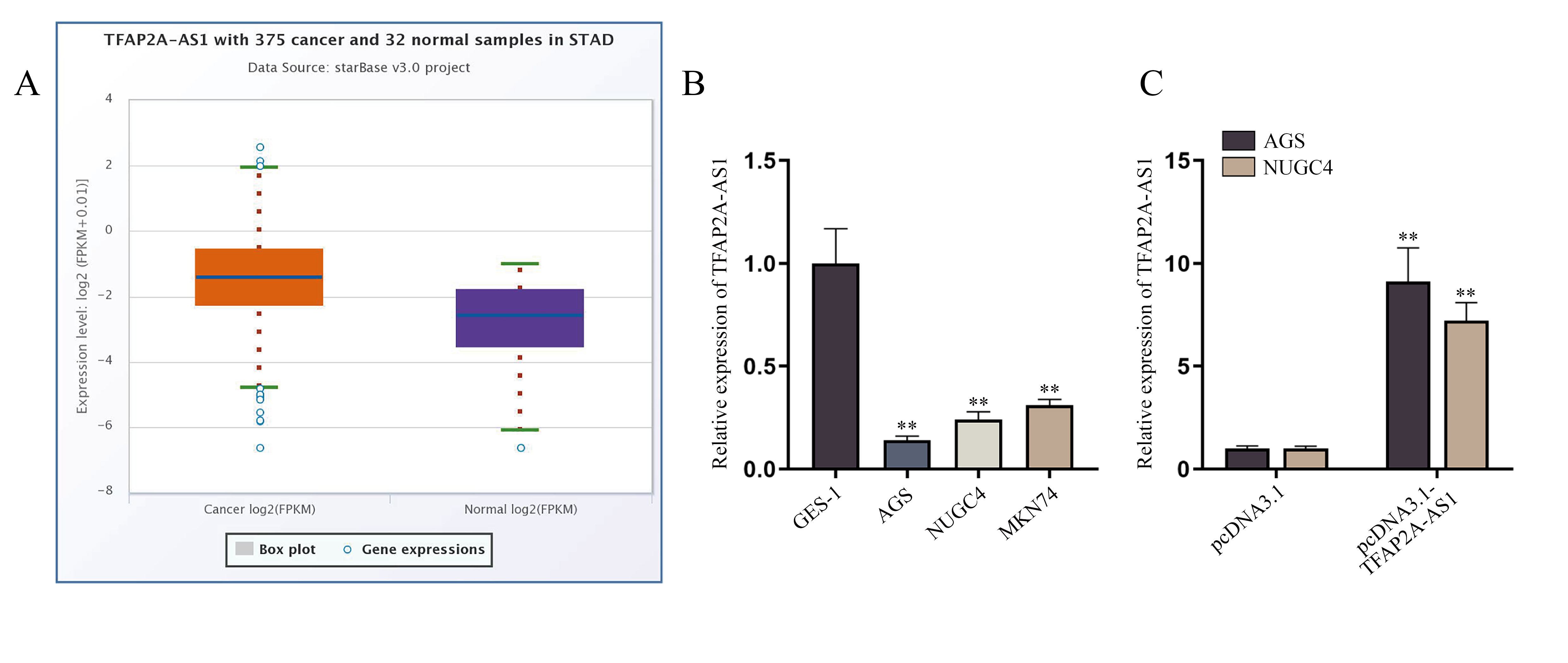

Supplement: Supplementary file 1 — Additional file 1: Fig. S1. (A) StarBase database (http://starbase.sysu.edu.cn/) was used to detect the expression of TFAP2A-AS1 in GC tissues and normal tissues. (B) The expression of TFAP2A-AS1 in GES-1, AGS, NUGC4 and MKN74 cells was detected by qPCR. (C) The overexpression efficiency of pcDNA3.1-TFAP2A-AS1 was detected by qPCR. The statistical analysis for Figure S1A and S1C was t-test, and for Figure S1B was two-way ANOVA. GAPDH was used as the internal reference for gene expression analysis. **P < 0.01. [file 13062_2021_300_MOESM1_ESM.tif]

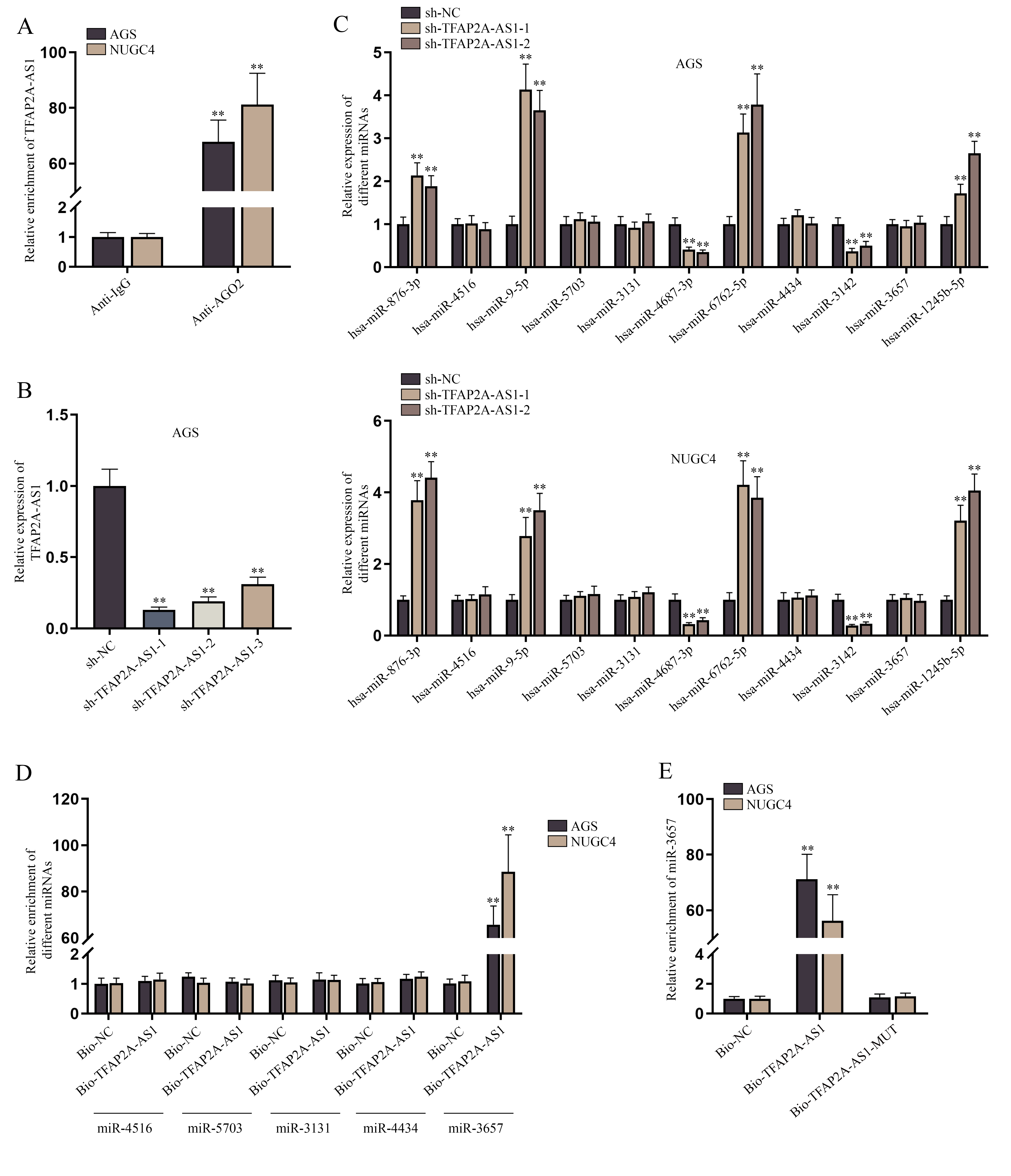

Supplement: Supplementary file 2 — Additional file 2: Fig. S2. (A) RIP assay was used to detect the enrichment of TFAP2A-AS1 in RISC of AGS and NUGC4 cells. (B) QPCR was used to detect the knockdown efficiency of sh-TFAP2A-AS1-1/2/3 in AGS cells. (C) QPCR was used to detect the expression of potential target miRNAs in AGS and NUGC4 cells after the knockdown of TFAP2A-AS1. (D) RNA-pulldown assay was used to detect the interaction of TFAP2A-AS1 with hsa-miR-4516, hsa-miR-5703, hsa-miR-3131, hsa-miR-4434 or hsa-miR-3657 in AGS and NUGC4 cells. (E) RNA-pulldown assay was used to explore the interaction between TFAP2A-AS1 and miR-3657 in AGS and NUGC4 cells. The statistical analysis for Figure S2A was student’s t-test, and for Figure S2B, S2C, S2D and S2E was one-way ANOVA. GAPDH was used as the internal reference for gene expression analysis. **P < 0.01. [file 13062_2021_300_MOESM2_ESM.tif]

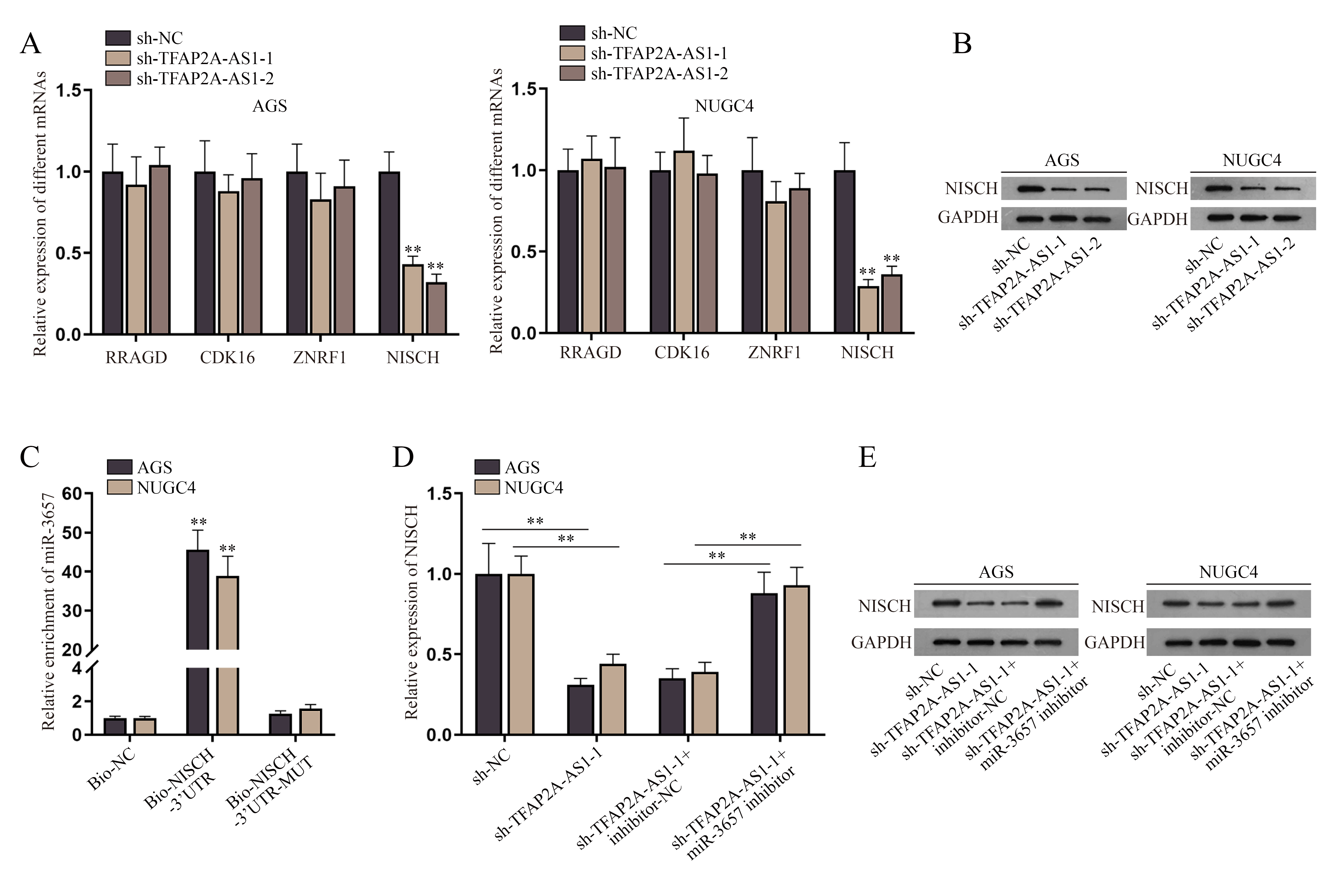

Supplement: Supplementary file 3 — Additional file 3: Fig. S3. (A) QPCR was used to evaluate the expression of potential target mRNAs after the knockdown of TFAP2A-AS1 in AGS and NUGC4 cells. (B) Western blot analysis was used to assess the protein level of NISCH in AGS and NUGC4 cells after the knockdown of TFAP2A-AS1. (C) RNA-pulldown assay was used to verify the interaction between NISCH and miR-3657 in AGS and NUGC4 cells. (D-E) QPCR and western blot analyses was used to detect the level of NISCH in AGS and NUGC4 cells after the transfection of sh-NC, sh-TFAP2A-AS1-1, sh-TFAP2A-AS1-1+inhibitor-NC or sh-TFAP2A-AS1-1+miR-3657 inhibitor. The statistical analysis for Figure S3A, S3C and S3D was one-way ANOVA. GAPDH was used as the internal reference for gene expression analysis. **P < 0.01. [file 13062_2021_300_MOESM3_ESM.tif]

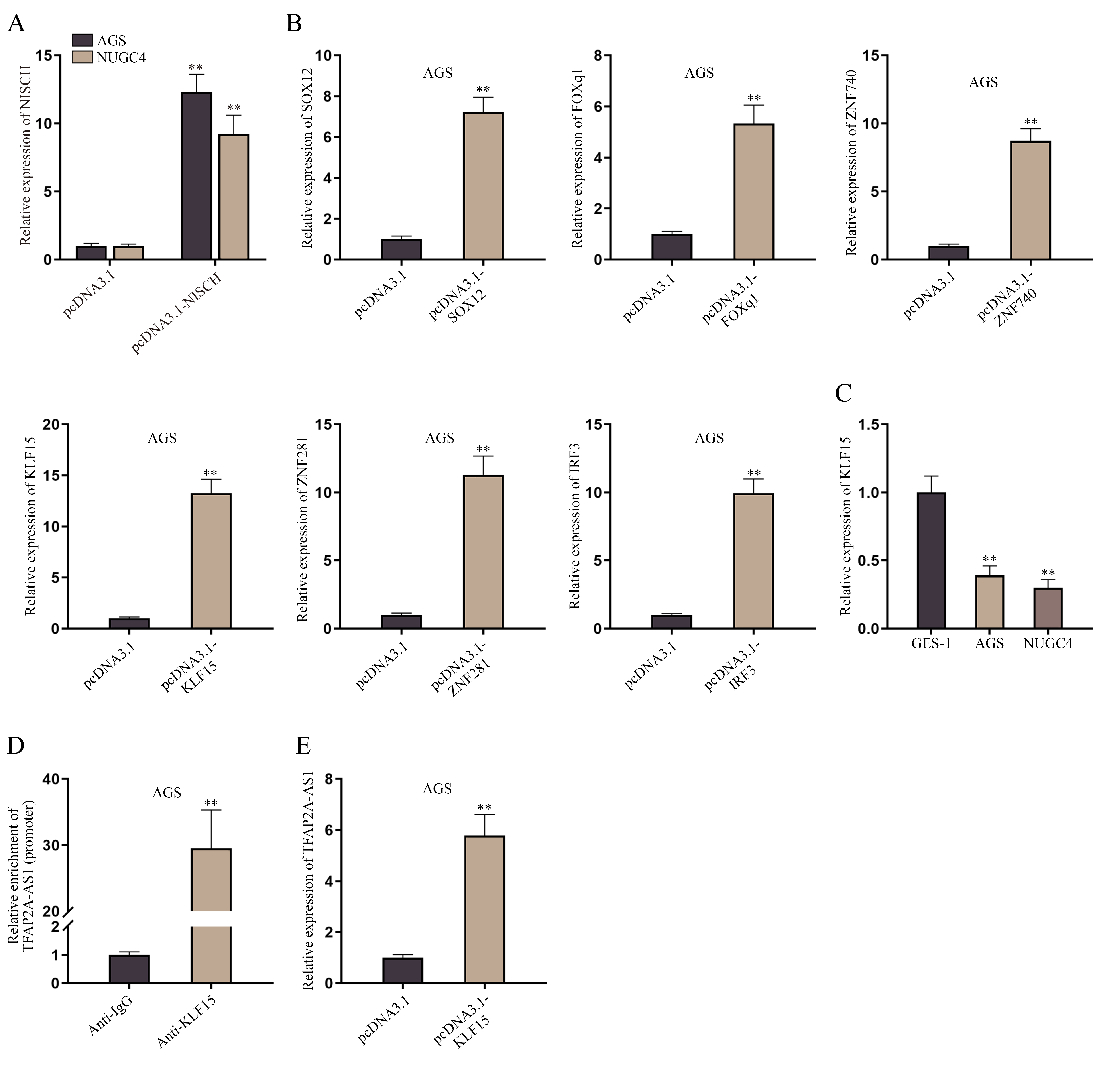

Supplement: Supplementary file 4 — Additional file 4: Fig. S4. (A) QPCR was performed to detect the overexpression efficiency of pcDNA3.1-NISCH in AGS and NUGC4 cells. (B) QPCR was used to evaluate the overexpression efficiency of pcDNA3.1-SOX12, pcDNA3.1-FOXq1, pcDNA3.1-ZNF740, pcDNA3.1-KLF15, pcDNA3.1-ZNF281 and pcDNA3.1-IRF3 in AGS cells. (C) QPCR was conducted to evaluate the expression of KLF15 in AGS, NUGC4 and GES-1 cells. (D) ChIP assays verified the interaction between TFAP2A-AS1 promoter and KLF15 in AGS cells. (E) QPCR detected the expression of TFAP2A-AS1 after the overexpression of KLF15 in AGS cells. The statistical analysis for Figure S4A, S4B, S4D and S4E was student’s t-test, and for Figure S4C was one-way ANOVA. GAPDH was used as the internal reference for gene expression analysis. **P < 0.01. [file 13062_2021_300_MOESM4_ESM.tif]
